# Serious lesions in Green turtles (*Chelonia mydas*) afflicted by fatal Spirorchiidiasis found stranded in south and southeastern Brazil

**DOI:** 10.1016/j.ijppaw.2023.01.004

**Published:** 2023-01-13

**Authors:** Hassan Jerdy, Bruna Barreto, Max Werneck, Rachel Ann Hauser-Davis, Paula Baldassin, Patrick Gabriel, Aline Luize de Moraes Souza, Maria Aparecida da Silva, Aline Felix, Rachel Ribeiro Rodrigues, Mariah Bianchi, Carla Barbosa, Gessica Gomes Vieira, Lara Ribeiro, Brenda Petronetto, Aline Souza, Renato Luiz Silveira, Eulogio Carvalho

**Affiliations:** a Laboratório de Microscopia, Universidade Federal do Sul e Sudeste do Pará, Rua Alberto Santos Dumont, Xinguara, PA, Brazil; b Laboratório de Morfologia e Patologia Animal (LMPA), Setor de Patologia Animal (SPA), Universidade Estadual do Norte Fluminense Darcy Ribeiro, Av. Alberto Lamego, 2000 – Parque Califórnia, Campos dos Goytacazes, Rio de Janeiro, Brazil; c BW Institute, Professora Sueli Brasi Flores Street, Number 88 Praia Seca, Araruama, RJ Zip Code (CEP), 28970-000, Brazil; d Laboratório de Avaliação e Promoção da Saúde Ambiental, Instituto Oswaldo Cruz (Fiocruz), 21040-360, Rio de Janeiro, RJ, Brazil; e Departamento de Biologia, Centro de Ciências Exatas, Naturais e da Saúde (CCENS), Universidade Federal do Espírito Santo (UFES), Alegre, ES, Brazil; f Instituto Argonauta, Brazil; g Departamentos de Morfologia (MMO), Patologia e Clínica Veterinária (MCV), Universidade Federal Fluminense (UFF), Niterói, RJ, Brazil

**Keywords:** Fatal spirorchiidiasis, Thyroid atrophy, Brain tissue loss, Splenic lymphoid depletion, Choroid layer destruction, Compression of air spaces

## Abstract

Several diseases have been reported as affecting endangered wild sea turtle population worldwide, including spirorchiidiasis. This parasitic infection results in serious circulatory disorders in sea turtles, as well as tissue damage due to the presence of spirorchiids eggs. However, few reports of organs severely affected by tissue replacement caused by granulomatous inflammatory processes due to spirorchiidiasis in sea turtles are available. In this regard, this study describes massive lesions in 16 juvenile green turtles from southeastern Brazil presenting no other detectable diseases or injuries, associated to parasitic compression of air spaces, parasitic thyroid atrophy, parasitic encephalic compression and parasitic splenic lymphoid depletion. These rare injuries were categorized as extremely severe, affecting most spirorchiidiasis-infected organs. Spirorchiidiasis was, thus, noted herein as capable of causing a variety of lethal injuries to vital or extremely important organs in sea turtles. Spirorchiidiasis should, therefore, also be considered a potential cause of death in stranded green sea turtle monitoring efforts.

## Highlights

• This study reports a new death cause in *Chelonia mydas.*
• Extremely severe atrophic lesions were observed.
• Lesions were compatible with inefficiency or malfunctioning of several organs.
• Brain compression and loss of brain mass was associated with large parasitic granulomas.
• This study demystifies the pathogenic potential of Spirorchiidae family parasites.

## Graphical abstract

**Figure:**
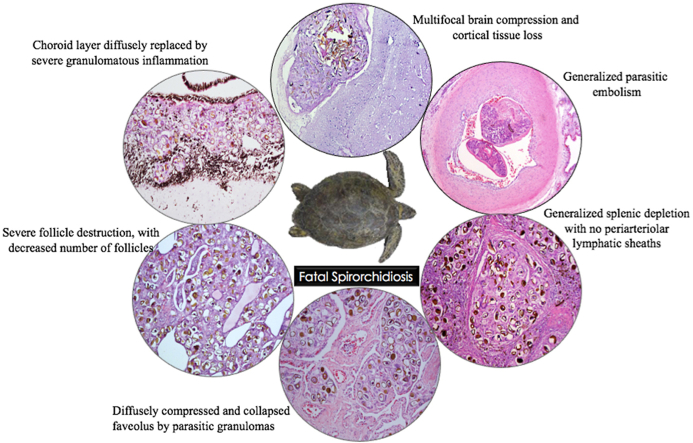

## 1 Introduction

Sea turtles exhibit a wide geographic distribution and are found in tropical to temperate seas and oceans worldwide. Most species are threatened with extinction, and several diseases have been reported as affecting endangered wild sea turtle population worldwide, mainly due to anthropogenic activities (Seminoff, 2004; Adnyana et al., 2020). These include fibropapillomatosis, ectoparasitism, and coccidiosis, and spirorchiidiasis, among others (see Adnyana et al. (2020) and references therein). However, infectious causes of turtle mortality, especially concerning spirorchiidiasis, are still poorly understood (Chapman et al., 2017).

Spirorchiidiasis infections are high in marine turtles, globally affecting endangered sea turtle populations, as the Spirorchiidae family is near cosmopolitan in its distribution (Chen et al., 2012; Chapman et al., 2017, 2019; Santoro et al., 2020). This parasitic infection has, in fact, been significantly associated to sea turtle stranding and mortality events worldwide (Santoro et al., 2007; Stacy et al., 2010; Werneck et al., 2015; Jerdy et al., 2020).

Spirorchiidiasis causes a wide range of inflammatory vascular reactions (Santoro et al., 2020). The lesions associated with this disease are caused by spirorchiids inhabiting the blood and lymphatic vessels of infected animals and by their egg deposition, known to affect most sea turtle organs (Santoro et al., 2007; Stacy et al., 2010). This condition has been reported as causing embolism, thrombosis, aneurysm, hypoxia, ischemia and arterial thickening (Stacy et al., 2010; Marchiori et al., 2017) and, as eggs are added to the infected vessels, larger clumps form and are surrounded by multinucleated giant cells, thus replacing part of organ parenchymas (Stacy et al., 2010). The detected granulomas can be classified according to the number of eggs and number of clumps in the analyzed tissue (Santoro et al., 2017).

Of the seven species of described sea turtles, five, namely *Chelonia mydas* (green sea turtle), *Eretmochelys imbricata* (hawkwbill sea turtle), *Caretta caretta* (loggerhead turtle), *Lepidochelys olivacea* (olive Ridley sea turtle) and *Dermochelys coriacea* (leatherback sea turtle), occur in Brazil. All are included in the National List of Endangered Species of Brazilian Fauna and are classified according to the International Union for Conservation of Nature (IUCN) as vulnerable, threatened or critically endangered (IUCN, 2022).

Sea turtle strandings in southeastern Brazil have been linked to chronic illness, endoparasites, and fibropapillomatosis as primary factors, although pollution has also been noted as an indirect threat, resulting in deleterious health effects and immunosuppression, leaving these animals more susceptible to opportunistic diseases (Tagliolatto et al., 2019). In this regard, although several cases of spirorchiidiasis in different hosts have been reported on the Brazilian coast (see Werneck et al., 2016), no cases of green sea turtles exhibiting lesions capable of causing severe functional losses in different organs but non-associated to other diseases have been reported so far in this region for this species. Thus, this study comprises a report on histopathological findings in sea turtles found stranded in south and southeastern Brazil with no other detectable diseases or injuries and presenting massive lesions due to spirorchiidiasis.

## 2 Material and methods

A total of 2600 green turtles found stranded through a beach monitoring program (Werneck et al., 2018), in the states of Paraná, São Paulo and Rio de Janeiro, in south and southeastern Brazil, were analyzed from November 2015 to March 2018. From this total, 740 individuals were infected with spirorchiidae eggs. The animals were all juvenile, with curvilinear carapace length ranging from 54 cm to 81 cm (Heppell et al., 2003; Reich et al., 2007) categorized according to Frazer and Ehrhart (1985). Of the 740 animals presenting, spirorchiidiasis, only turtles with no other detectable mild or severe diseases or injuries and severely affected by spirorchiidiasis (see Santoro et al., 2017) were selected. In addition to other organs, all animals presented spirorchiidiasis in the lungs, thyroid, brain, ocular bulb, and spleen in varying degrees.

Tissue samples were obtained during the necropsies, fixed in 10% neutral buffered formalin for 48 h and analyzed at the Laboratory of Animal Morphology and Pathology at the Norte Fluminense State University, in Rio de Janeiro, southeastern brazil. Routine sample processing was conducted, obtaining serial 5 μm sections of the following organs: adrenals, spleen, brain, cerebellum, heart, esophagus, stomach, liver, salt gland, eye bulb (cut for good fixation), small intestine, large intestine, tongue, skeletal striated muscle, bone marrow, spinal cord, ovary, pancreas, parathyroid, skin, choroid plexus, lung, kidney, adipose tissue, thymus, thyroid, trachea, testis, gallbladder, urinary vesicle. Fifteen serial histological sections were obtained from compromised spleens, in the middle (five sections) and on the spleen edges (five sections each edge) to evaluate periarteriolar lymphocyte sheaths. Histological nomenclature followed Zhang et al. (2016). To assess focal cerebral granuloma compression, a total of 23 granulomas in the pia mater affecting the cerebral cortex of nine turtles with severe cerebral spirorchiidiasis were assessed. Compression was determined by comparing measures between preserved tissue surrounding severe granulomas and within the granulomas. In the lungs, the thickness of 50 interfaveolar septa with (N = 25 septa) and without (N = 25 septa) granulomas were randomly measured in 11 individuals, totaling 550 measured septa. In the thyroid, 1265 thyroid follicles were counted from the serial sections of 11 animals displaying severe thyroid damage to quantify empty and weakly-to strongly-stained lumens. All analyses were performed using a Nikon Eclipse 80i microscope (Kurobane Nikon Co., Ltd, Otawara, Tochigi, Japan) employing the NIS Elements BR software program.

Egg types were assessed based on Wolke et al. (1982), where type 1 eggs are elongated and have polar processes and some have a hook-shaped tip, type 2 eggs are oval and have a polar process and type 3 eggs are rounded without polar processes.

All turtle data (size, nutritional condition, examination details, spirorchiidae egg types and lesion severity, clinical history and basis for cause of death determination are exhibited in Table 1.

**Table 1:** Turtle ID, size, nutritional condition, examination details, spirorchiidae egg types and lesion severity, clinical history and basis for cause of death determination.

ID	Size	Nutritional Condition	Details of examination	Egg type and lesions severity	Clinical history	Basis for cause of death determination
1	61 cm	Cachectic	Evident bones	Eggs type 1 (severe)	Animal found floating, apathetic, unresponsive to visual and food stimuli.	Animal with severe and generalized spirorchidiasis and no gross or histopathological changes consistent with other infectious agents, drowning, necrosis or inflammations of any degrees.
2	57 cm	Cachectic	Evident bones	Eggs type 1 (severe)	Animal found stranded dead.	
				Eggs type 3 (severe)		
3	78 cm	Thin	Seaweed in carapace	Eggs type 1 (severe)	Animal found floating, responsive to food stimuli.	
				Eggs type 3 (severe)		
4	64 cm	Cachectic	Evident bones	Eggs type 1 (severe)	Animal stranded alive on the beach, responsive to food stimuli.	
5	77 cm	Cachectic	Common marks on plastron and evident bones	Eggs type 1 (moderate)	Animal found floating alive, unresponsive to visual and food stimuli.	
				Eggs type 3 (severe)		
6	71 cm	Cachectic	Evident bones	Eggs type 1 (severe)	Animal found stranded dead.	
				Eggs types 2 (moderate)		
				Eggs type 3 (severe)		
7	54 cm	Cachectic	Evident bones	Eggs type 1 (severe)	Animal found stranded alive by the monitoring team.	
				Eggs type 3 (severe)		
8	61 cm	Cachectic	Evident bones	Eggs type 1 (severe)	Animal found stranded dead.	
				Eggs type 3 (severe)		
9	76 cm	Cachectic	Evident bones	Eggs type 1 (severe)	Animal stranded alive, unresponsive to food stimuli.	
10	55 cm	Thin	Evident bones	Eggs type 1 (moderate)	Animal found stranded dead.	
				Eggs type 3 (severe)		
11	81 cm	Cachectic	Evident bones	Eggs type 1 (severe)	Animal found alive floating, unresponsive to visual and food stimuli.	
				Eggs type 3 (severe)		
12	79 cm	Cachectic	Evident bones	Eggs type 1 (severe)	Animal found stranded dead.	
13	58 cm	Cachectic	Evident bones	Eggs type 1 (severe)	Animal found stranded dead.	
				Eggs type 3 (severe)		
14	69 cm	Cachectic	Evident bones	Eggs type 1 (severe)	Animal found stranded alive on the sandy beach.	
15	62 cm	Cachectic	Evident bones	Eggs type 1 (severe)	Animal found stranded dead.	
				Eggs type 3 (severe)		
16	74 cm	Cachectic	Evident bones	Eggs type 1 (severe)	Animal found stranded alive in the sand, unresponsive to visual and food stimuli.	
				Eggs type 3 (severe)		

## 3 Results

Egg granulomas belonging to the spirorchiidae family were observed in all 16 green turtle specimens, with various levels of severity in the following organs and tissues: adrenal, spleen, brain, cerebellum, heart, esophagus, stomach, liver, salt gland, eye bulb (choroid layer), small intestine, large intestine, tongue, skeletal striated muscle, bone marrow, spinal cord, ovary, pancreas, parathyroid, skin, choroid plexus, lung, kidney, adipose tissue, thymus, thyroid, trachea, testis, gallbladder, urinary vesicle.

Histopathologic examinations of these tissues indicated that all green turtle individuals presented embolism associated with parasites and eggs. Adult parasites were detected the heart and vessels (pia mater, pulmonary, choroid layer of the eye bulb, salt gland, intestinal serosa, splenic and adrenal). Embolisms were detected in the lung, intestine, spleen, adrenal, liver, salt gland, kidney, eye bulb, brain, thyroid pancreas, caused by parasite eggs and the parasites themselves, as well as arteritis and parasitic granulomatous phlebitis.

### 3.1 Respiratory system

**Fig. 1:**
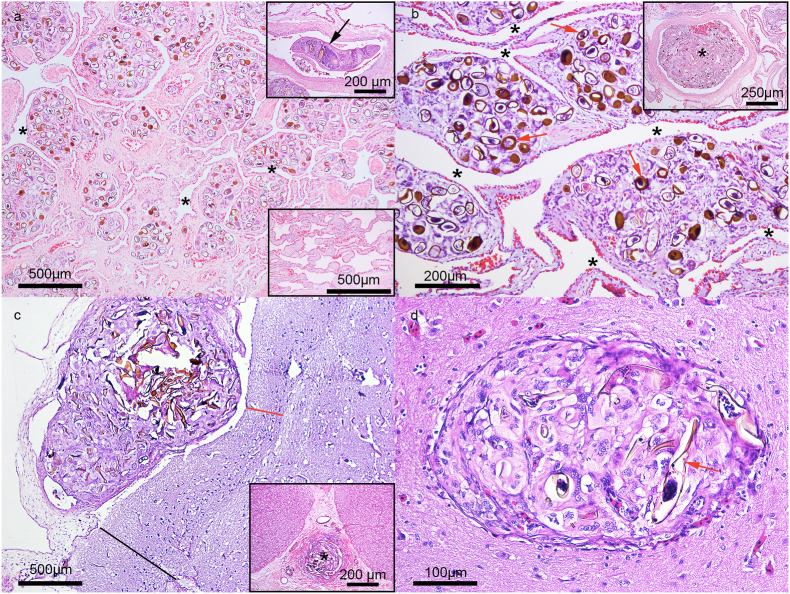
a. Severe and Generalized Granulomatous Interstitial Pneumonia, Lung, *C. mydas*. Lung tissue extensively affected by large parasitic multifocal to coalescent severe granulomas in interfaveolar septa with diffusely compressed and collapsed faveolus (*). Upper Inset: Parasite of the spirorchiidae family in pulmonary artery lumen. Bottom Inset: Lung. Normal *C. mydas* lung. Fig. 1 b. Severe and Generalized Granulomatous Interstitial Pneumonia, Lung, *C. mydas*. Higher magnification of severely enlarged interfaveolar septa with diffusely compressed and collapsed faveolus (*), note egg type 3 (red arrow). Inset: Thrombus formed by eggs, cellular debris, macrophages and multinucleated giant cells in artery. Fig. 1 c. Granulomatous Meningitis, Brain, *Chelonia mydas*. Parasitic granulomas associated with severe nervous tissue atrophy associated with cerebral cortex loss, note red line with 290 μm (cerebral cortex compression area) and black line (722 μm) without cerebral cortex compression. Inset: Embolism, Spinal Cord. Embolus formed by cluster of eggs (*) in arteriole. Fig. 1 d. Brain, Granulomatous Encephalitis, *Chelonia mydas*. Parasitic granulomas associated with neural parenchyma compression, note egg type 1 (red arrow), (hematoxylin and eosin staining). (For interpretation of the references to colour in this figure legend, the reader is referred to the Web version of this article).

The lungs of 11 animals exhibited a severe diffuse coalescent granulomatous interstitial inflammatory process characterized by large clumps of type 1, 2 and 3 parasite eggs, surrounded by reactive macrophages and multinucleated giant cells with rare lymphocytes in interfaveolar septa with thin reactive fibrous tissue. The pulmonary faveolus was severely and diffusely collapsed with a marked decrease in the faveolar lumen (Fig. 1a). These lesions are compatible with severe granulomatous interstitial pneumonia and severe pulmonary atelectasis caused by parasites. The thickness of the interfaveolar septa without granulomas ranged from 24 to 78 μm, while the interfaveolar septa with granulomas ranged from 306 to 687 μm. The 225 interfaveolar septa presenting inflammation were 10.5 x thicker than healthy septa (Fig. 1b). The eggs found in the lungs are compatible with the Hapalotrema, Learedius, Monticellius, Amphiorchis, Carettacola and Neospirorchis genera.

### 3.2 Central nervous system

The pia mater of nine animals presented lesions composed of granulomas displaying a thin pseudocapsule and severe focal brain compression. The granuloma consisted of many type 1 and 3 parasite eggs surrounded by multinucleated giant cells with a central area of caseous necrosis. The 23 pia mater granulomas compressed the molecular layer of the telencephalon with an average of 423 μm of tissue compression, determining parasitic cerebral compression associated with atrophy and brain tissue loss (Fig. 1c). One animal displayed large multifocal parasitic granulomas in the pia mater and gray matter (Fig. 1d). Parasites in the lumen of arterioles of the pia mater were also detected. The eggs found in the brain are compatible with the Hapalotrema, Learedius, Monticellius, Amphiorchis, and Neospirorchis genera.

### 3.3 Endocrine system

**Fig. 2:**
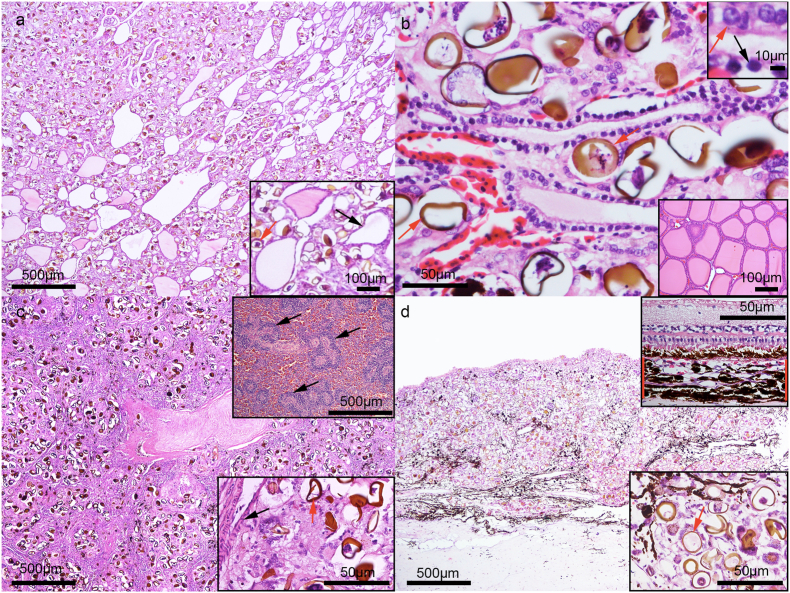
(a–b). Granulomatous Thyroiditis, Thyroid, *C. mydas*. Figure a. Severe follicle destruction, with decreased number of follicles. Figure a. Inset: Parasitic granulomas associated with compressed, deformed, empty follicle (arrow). Figure b. Upper Inset: Atrophic thyroid follicles with normal epithelial cell (red arrow) and randomly pyknotic follicular cells (black arrow). Additionally note a type 3 egg (red arrow). Bottom Inset: Thyroid, Normal *C. mydas* thyroid. Figure c. Granulomatous Splenitis, Spleen, *C. mydas*. Large and severe coalescent granulomas associated with marked and diffuse lymphoid depletion and periarteriolar lymphatic sheaths loss. Upper Inset: Spleen. Normal *C. mydas* spleen, note periarteriolar lymphatic sheaths (black arrow). Bottom Inset: Higher magnification of granulomatous splenitis associated with periarteriolar lymphoid depletion, note arteriole (black arrow) and type 3 egg (red arrow). Figure d. Granulomatous Choroiditis, Ocular Bulb, *C. mydas*. Choroid layer diffusely replaced by severe granulomatous inflammation. Upper Inset: Choroid layer and retina. Normal *C. mydas* choroid layer (between red lines) and retina. Bottom Inset: Higher magnification of severe granulomatous inflammation associated with egg type 3 (red arrow), (hematoxylin and eosin staining). (For interpretation of the references to colour in this figure legend, the reader is referred to the Web version of this article).

Thyroid lesions in eleven animals consisted of extensive masses formed by sometimes “bridged” coalescent granulomas that diffusely contoured, dissociated and replaced thyroid follicles. Granulomas consisted of massive clumps of type 1 and 3 parasite eggs, surrounded by multinucleated giant cells and activated macrophages that replaced sparse diffusely compressed, deformed, ruptured thyroid follicles. The colloid was weakly stained or exhibited empty follicular lumen (Fig. 2a), with follicular cells sometimes pyknotic with a clear perinuclear cytoplasmic halo (Fig. 2b). Form a total of 1265 thyroid follicles, 726 presented an empty lumen and 539 exhibited a weak to strongly stained lumen, with an average of 57.3% of follicles with an empty lumen. In *foci* displaying less severe inflammation processes, follicles were dissociated by the formation of coalescing granulomas by “bridging granuloma” parasite eggs. The eggs found in the thyroid are compatible with the Hapalotrema, Learedius, Monticellius, Amphiorchis, and Neospirorchis genera.

### 3.4 Lymphoid system

The spleen lesions in 12 turtles consisted of a severe granulomatous inflammatory process that markedly replaced the white and red pulps of this organ. The serial sections exhibited no preserved periarteriolar lymphatic sheaths, and the lymphoid tissue was sparse and severely dysplastic. Large granulomas were observed around the ellipsoid and central arteries, most of the time coalescing and involved by thin to moderate reactive fibrous tissue. The granulomas were caused by type 3 parasite eggs occupying and severely replacing white and red spleen pulp (Fig. 2c), compatible with lymphoid depletion associated with a severe parasitic granulomatous process. Parasites occluding the lumen of arterioles (embolism) were also observed in sick animals. The eggs found in the spleen are compatible with the Neospirorchis genera.

### 3.5 Ocular bulb

The choroid layer of 14 animals presented extensive coalescent to diffuse lesions, composed of marked granulomatous inflammation in the choroid layer with complete blood vessel destruction, replacement, and retinas were detached in all animals (Fig. 2d). Inflammation was composed of a marked number of multinucleated giant cells and macrophages involving the observed type 1 and 3 parasite eggs, with a moderate number of lymphocytes, plasma cells and rare heterophils. The stroma was reactive, represented by loosely organized fusiform to stellate fibrocytes and fibroblasts. Fibrocytes exhibited moderate amounts of eosinophilic vacuolated cytoplasm associated with rare blood capillaries and randomly distributed reactive melanophores. The eggs found in the brain are compatible with the Hapalotrema, Learedius, Monticellius, Amphiorchis, and Neospirorchis genera.

### 3.6 Parasites

Spirorchiid type 1 eggs were observed in the tissues of all 16 animals, with severe lesions, associated to the *Learedius*, *Monticellius*, *Amphiorchis and Hapalotrema* genera. Type 2 eggs are characteristic of the *Carettacola* genus and were observed in only one green sea turtle in this study. Type 3 eggs were observed in 11 individuals and are characteristic of the *Neospirorchis* genus.

Additionally, no bacterial colonies, other parasitoses, protozoa or mild lesions compatible with viruses, such as random necrosis or viral inclusion bodies were present in any of the analyzed turtles.

## 4 Discussion

Interfaveolar septa 10 x thicker than normal and displaying a diffuse form was observed as a form of severe compression of air spaces caused by parasites in the affected animals, capable of causing systemic hypoxia. This condition can cause severe damage to cardiac myocytes, hepatocytes, neurons, and renal tubular cells, as well as death (Zachary, 2018). In addition to loss of respiratory function, several blood vessels in these animals were partially to fully obstructed by parasitic emboli and thrombi, making organ oxygenation even more difficult. Several articles cite pneumonia in severe cases (Gordon et al., 1998), but the suggestion of loss of respiratory capacity in severe cases of pulmonary spirorchidiasis has never been postulated, perhaps because extremely severe cases such as those in this study are rare and organs were massively replaced by large coalescing granulomas.

Neurological deficits were only noted antemortem in turtles with severe neurospirorchiidiasis. Lesions in the brain parenchyma were largely mild to moderate, with usually more severe and, in some cases, diffuse lesions noted within the meningeal compartment, (Flint et al., 2010). Severe brain compression is a serious undescribed injury that causes neuronal dysfunction by preventing normal axon antegrade and retrograde flows. It can also result in reduced blood flow to the nerves, contributing to neuronal dysfunction. Gentle compression can result in partial blockage of slow axoplasmic flow and gradual neurofilament and microtubule accumulation, resulting in mild degeneration. Eventually, the distal axon is lost during long periods of severe compression with axoplasmic blockage (Zachary, 2018). In the present study, nine animals exhibited long-term multifocal compression caused by large granulomas and loss of the cortical region of the telencephalic nervous tissue. Neurospirorchiidiasis has been well reported in other seau turtle species, such as *Caretta caretta*, associated with type 3 eggs (Jacobson et al., 2006), in contrast with the present study, in which most turtles were associated with type 1 eggs, possibly due to different species habits.

Empty follicles were inactive, exhibiting colloid vacuolization, indicating colloid reabsorption. These findings indicate decreased thyroid gland activity (Lee et al., 2016). This is the first report of thyroid atrophy for sea turtles, which, in other animals, generates numerous functional disorders associated with hypothyroidism (Zachary, 2018). Although no hypothyroidism was detected herein, a suggestive functional loss of the thyroid is noted, based on follicular necrosis, with a consequent decrease in the number of follicles, alongside compression and colloid absence in most lumens. Compressed, with little or no colloid (Stacy et al., 2010). The observed follicular necrosis, resorption and colloid non-production detected in most of the investigated thyroid follicle lumens are probably associated to a lack of adequate blood supply. Thyroids exhibited intense pleocellular infiltrate, granulomatous inflammation, thrombosis, and loss of thyroid follicles. This is, to the best of our knowledge, the only article describing described a lesion other than thyroiditis in sea turtles, although loss of colloid, empty follicles and thyroid atrophy have not yet been reported for this taxon.

Although sea turtles do not have lymph nodes, the spleen is not the only lymphoid organ, as a thymus is also present (Wyneken, 2001). The spleen is the main monocytic-macrophagic system site, responsible for filtering the blood through microorganism phagocytosis and playing a role in the immune response (Zachary, 2018). Herein, the most affected spleen sites were located around the central and ellipsoidal arterioles, where the periarteriolar lymphocyte sheaths are located, which were severely and diffusely destroyed and replaced in several histological sections. Lymphoid depletion directly impairs the foreign antigen capture and phagocytosis in the blood and antigen presentation to T-lymphocytes, which, in turn, lead to T- and B-lymphocyte activation and antibody production. This condition facilitates the proliferation of various microorganisms that can may cause sea turtle death (Zachary, 2018). The absence of lymph nodes may make these animals more susceptible to immunoweakness due to diffuse severe splenic spirorchiidiasis. Spleen with multifocal granulomas, large granulomas (severe lesions) have been reported previously (Stacy et al., 2010; Chapman et al., 2017; Santoro et al., 2017). However, lymphoid depletion and large coalescing granulomas throughout the entire parenchyma were only reported in the present study.

Severe ocular spirorchiidiasis is associated to a complete loss of choroidal layer function, tissue architecture, and normal thickness in many cases (Jerdy et al., 2020). The 16 animals analyzed herein presenting severe ocular spirorchiidiasis exhibited retinal detachment, so it is possible that the reduced granuloma blood supply is not sufficient to maintain retinal nutrition. This condition has been reported previously for different sea turtle species in southeastern Brazil, postulated as resulting in partial or total loss of sight (Jerdy et al., 2020) and, probably, the main cause for the cachectic state of most animals displaying this condition, as loss of vision probably results in inadequate foraging activities. It is important, however, to note that, retinal detachment is a common postmortem artifact in the eyes of sea turtles. Unfortunately, no retinas were available for analysis to confirm real lesions in the retinal epithelium, requiring further associations to spirorchiidae infection for more concrete conclusions in this regard.

Spirorchiid type 1 eggs were observed in the tissues of all 16 animals, with severe lesions, associated to the *Learedius*, *Monticellius* and *Amphiorchis* genera. Type 2 eggs are characteristic of the *Carettacola* genus and were observed in only one green sea turtle. Type 3 eggs were observed in 11 individuals and are characteristic of the *Neospirorchis* genus (Wolke et al., 1982). Although the present study did not prioritize parasite collection from the hosts, the presence of spirorchiid eggs and their respective genera corroborate previous sea turtle findings in the states of Rio de Janeiro and São Paulo (Werneck et al., 2016).

## 5 Conclusion

Sixteen severe spirorchiidiasis cases in green sea turtles are reported herein, resulting in various extremely debilitating conditions, incompatible with life, without any other associated injuries or illnesses. Severe granulomatous pneumonia associated with severe and diffuse severe compression of air spaces can interfere with gas exchanges, potentially causing hypoxia. Thyroid lesions are compatible with considerable thyroid activity decreases. Severe splenic lymphoid depletion can compromise immunity and responses to infectious agents. Multifocal brain compression may result in severe neuronal loss and function. Severe ocular spirorchiidiasis is associated with decreased nutrition and potential retinal detachment. In addition to the risk of parasitic septic shock, the pathophysiology described in this assessment allows us to conclude that spirorchiidiasis is capable of causing a variety of lethal injuries to vital or extremely important organs. Injuries of this severity are serious, and so far, have been unreported or neglected, as no published reports containing lesion images and injury consequences as severe as those reported herein are available. Spirorchiidiasis should, therefore, also be considered a cause of death in stranded green sea turtle monitoring efforts, as this study indicates that spirorchiidiasis lesions can be even more severe than stated by most reports.

## Declaration of competing interest

None.
